# Optimization of prodigiosin biosynthesis by *Serratia marcescens* using unconventional bioresources

**DOI:** 10.1186/s43141-020-00045-7

**Published:** 2020-07-09

**Authors:** Ashlesha Bhagwat, Unnati Padalia

**Affiliations:** 1 Department of Food Engineering and Technology, Institute of Chemical Technology, Mumbai, 400019 India; 2 Department of Microbiology, K. J. Somaiya College of Science and Commerce, Mumbai, 400019 India

**Keywords:** *Serratia marcescens*, Oil seed cakes, Optimization, Prodigiosin, Pigment, GC-MS

## Abstract

**Background:**

Prodigiosin is a naturally occurring red pigment by *Serratia marcescens* and having enormous medicinal properties. Recently, there is a need to develop a high-throughput and economically feasible bioprocess for the production of prodigiosin. In order to find a cost-effective alternative to individual fatty acids as substrate in industries, we tried to study the effect of different fatty acid containing oil seed cakes of peanut, sesame, and mustard as sources of substrate. The present study screened waste and unconventional bioresources for the production of prodigiosin using *S. marcescens* ATCC 13880. Sources with high oil content were screened for maximum production of prodigiosin. Also, various parameters like temperature, pH, and nutrient precursors were screened and optimized for the production of prodigiosin.

**Results:**

Scaled-up of optimized media consisting of 4% peanut oil seed cake powder, 2% sucrose, pH 7.5, temperature 28 °C, and 72 h incubation time resulted in highest production of 15.5 g/L wet biomass and 0.9 g/L of dried prodigiosin. Further, UV scan of the pigment showed maximum absorbance at 538 nm which is physiological property of the pigment. Extraction and purification of the pigment at the commercial level using the chromatographic techniques and mass spectral analysis confirmed the presence of prodigiosin.

**Conclusion:**

Using oil-extraction leftover wastes might help in the commercial and cost-effective production of prodigiosin.

## Background

Prodigiosin is a secondary metabolite widely produced by several species including *Serratia marcescens*, *Vibrio psychroerythrous*, *Pseudomonas magneslorubra*, and other such bacteria [1]. This multifaceted secondary metabolite is a red pigment with a common 4-methoxy-2,2 bipyrrole ring system and has a series of close relatives bearing the same structure with different alkyl substituents [2]. Prodigiosin is known to have immunosuppressive, anti-fungal, anti-viral, anti-microbial, anti-malarial, and anti-proliferative properties. It also induces apoptosis in cancer cells [3, 4]. The anti-bacterial activity of prodigiosin is attributed to its ability to pass through the outer membrane thereby inhibiting enzymes, like DNA gyrase and topoisomerase IV involved in cell growth [5]. There are reports that have revealed prodigiosin’s ability of suppressing graft versus host disease in mouse models as well as prevention of collagen-induced arthritis in mouse models [6].

Pigments are widely used in manufacturing paints, ink, plastics, fabrics, cosmetics, food, etc. Due to technology development, synthetic pigments are produced and used in the fabric industry. Recently, there is a high demand for natural pigments. Natural pigments are mainly obtained from plants and microorganisms. They produce more eco-friendly and sustainable pigments. Microorganisms have been screened and reported to produce secondary metabolites in form of pigments, like carotenoids, melanins, flavins, and phenazines. The toxic nature of artificial pigments have rendered a high demand for microbial pigments which are safer to use [7].

*Serratia*, a Gram-negative organism has widely spread sources such as air, water, soil, plants, fruits, and animals [8]. They are prolific producers of the pigment “prodigiosin.” Several reports have suggested use of typical complex media for fermentative production of prodigiosin by *S. marcescens* that are rich in a variety of nutrients [9, 10]. Some nutrients, like thiamine and ferric acid [11], are crucial for enhanced prodigiosin production, while phosphate [12], adenosine triphosphate, and ribose [13] have shown opposite effect by inhibition of prodigiosin yield. Aniyan and Thomas have suggested use of solid-state fermentation using less liquid medium for pigment production in *Serratia marsescens*. The solid state is known to give high nutrient concentration and availability from the substrate for pigment production [8]. Also, various parameters, like temperature, incubation time, pH, nutrient source, and quorum sensing, are the main factors that have been reported to impact prodigiosin production [14].

Owing to its medicinal and industrial properties, there is a demand to develop high-throughput and cost-effective bioprocesses for prodigiosin production. Selection of inexpensive raw materials is crucial for economically feasible production process of prodigiosin and that accounts for 50% of the total product cost. The rapid increase in industrial waste has developed a need to convert this waste to a value added product with an eco-friendly approach [15]. Traditionally, fats, oils, glycerol, and carbohydrates with hydrocarbons and non-hydrocarbon content are used for the growth of pigment producing microorganism [16, 17]. Giri et al. had screened a series of media and discovered that media containing peanut seed and sesame seeds gave significant enhancement of prodigiosin production [1]. Oil seed cakes have been reported to be an excellent source of nutrients, like protein, minerals, crude fibre, and fatty acids, and have also been studied as food supplements [18].

In the present study, we aimed to produce prodigiosin from natural, inexpensive, and eco-friendly carbon sources. We screened oil seed cakes from oil factories that had been left after the oil extraction process. These sources included peanut, mustard, and sesame seed oilcakes as a source of unconventional economical media for prodigiosin production.

## Methods

Peanut seed, mustard seed, and sesame seed oil cakes were purchased from a local oil factory located in Mumbai. All the chemical and reagents used were of AR grade and purchased from Himedia, Mumbai. *S. marcescens* ATCC 13880 was a gift sample from K. J. Somaiya Microbiology Department, Mumbai. The culture was maintained on Luria Bertanni (LB) agar medium and sub-cultured after every 15 days. The slants were stored at 4 °C in a refrigerator.

### Prodigiosin production and its estimation by UV-Vis spectroscopy

In order to compare pigment production, both liquid and solid forms of media were screened for pigment production. Briefly, 20 ml of nutrient broth (NB) was inoculated with fresh 5% culture. Similarly, nutrient agar (NA) plate was spread with 5% of culture. Both the media were incubated for 72 h and evaluated for pigment production. The quantitative determination of the red pigment was done by measuring the absorbance at 530 nm using double beam UV-Visible spectrophotometer as suggested by Chen et al. [17]. In case of solid medium, the culture was scraped and suspended in 20 ml distilled water (D/W). For the extraction process, 5 ml from above broth was taken in a test tube, and 4 ml of methanol was added. The mixture was vigorously vortexed for 2 min. The solution was then centrifuged for 10 min at 6000 rpm. Following the above step, 0.8 ml supernatant was further mixed with 0.2 ml of 0.05 N HCl: methanol mixture (4:1 v/v). The absorbance of the resulting solution was then measured at 530 nm [17].

### Optimization of culture growth conditions for prodigiosin production

Sterile nutrient agar was used as the basal medium, as the culture failed to grow in broth. Important parameters, like pH (6.5, 7, 7.5, and 8), temperature (25, 30, and 37 °C), and incubation time (24, 48, 72 h) were checked for optimum pigment production.

The effect of carbon source (each 2% glucose, sucrose, lactose, and fructose), nitrogen source (1% of peptone, yeast extract, beef extract, casein hydrochloride) were also studied, respectively. After optimizing each parameter for maximum prodigiosin production, the same was selected and maintained throughout the study.

For optimization of fatty acid sources, natural substrates, like oil seed cakes of peanut, mustard, and sesame, were crushed in a mixer and sieved to fine particles. Fine particles of oil seed cakes were stored at 4 °C until further use. Nutrient agar supplemented with 2% oil seed cake powder was used for prodigiosin production using *S. marcescens* strain. The pH was adjusted to 7.5 using 1 N NaOH. All the media were autoclaved at 121 °C for 20 min. All experiments were carried out in triplicates. The unconventional sources which gave higher production of prodigiosin such as peanut and sesame seed powder were further screened for their concentration in the range from 1 to 4%.

### Effect of solvents in prodigiosin extraction

Solvents, like ethanol, methanol, acetone, and dimethyl sulfoxide (DMSO), were used to extract crude prodigiosin. Briefly, the culture was scraped from Petri plates and washed with 20 ml of D/W. The washed pellet was dissolved in 4 ml each of the mentioned solvents and absorbance reading was noted at 530 nm. Higher absorbance reading exhibited higher pigment extraction.

### Scale-up studies for prodigiosin biosynthesis and biomass estimation

After optimization of all the parameters, prodigiosin biosynthesis was determined by upscaling the protocol using 1 L media. The medium was prepared using optimized parameters, like 4% peanut oil seed cake powder, 2% sucrose, 1% casein hydrolysate, pH 7.5, temperature 28 °C, and incubated for 72 h. Briefly, the culture was scraped off from Petri plates and suspended in 20 ml of D/W. The cells were centrifuged at 10 min at 6000 rpm and suspended in D/W, vortexed, and centrifuged again to obtain pellet. The pellet was dissolved in 4 ml of methanol, vortexed, and centrifuged again. The resulting pigmented supernatant was collected by filtering through 0.2 μ Whattman filter paper. The filtrate obtained was dried in a hot air oven at 55 °C overnight. The crude powder was again suspended in 4 ml of chloroform and dried again. The obtained powder was then weighed and expressed as gram per liter.

### Purification, confirmation, and characterization of pigment

In this study, the pigment pellets were collected from 1 L media by scrapping the Petri plates. The washed pellets were then dissolved in 50 ml of acidified methanol (4 ml + 96 ml methanol) followed by ultrasonication for 30 min. The supernatant was left to stand for 1 h. The supernatant was then collected by rotary evaporation to obtain the crude form of the pigment. The crude form of pigment was then purified using a silica gel column. The pigment was eluted using petroleum ether for five volumes followed by petroleum ether: ethyl acetate (1:1 v/v). The red elute was collected and concentrated using rotary evaporator at 40 °C [19].

The pigment was confirmed by UV wavelength scan, thin layer chromatography, and Gas chromatography-mass spectrophotometry. Two purified pigment extracts (3 ml) were separately dissolved in methanol and dimethyl sulphoxide (DMSO) solvents and scanned under UV-spectrophotometer (Beckman DU-800) in the range 200–800 nm for maximum absorbance.

The purified pigment extracts dissolved in methanol and DMSO separately were then characterized using subjected thin layer chromatography using a developing solvent (70% acetone + 30% hexane). The *R*_f_ values were compared to values reported in the literature. The pigment extract was dissolved in 5 ml methanol and subjected to mass spectral analysis (GC-MS) to determine the molecular mass of the pigment by comparing prodigiosin derivatives as reported in the literature. Vitamin A, a chemical analog of prodigiosin with similar pyrrole ring structure, molecular weight (333 amu), was used as a reference compound. The mass match was carried out by a computerized search with already registered prodigiosin-like compounds [20].

## Results

**Fig. 1:**
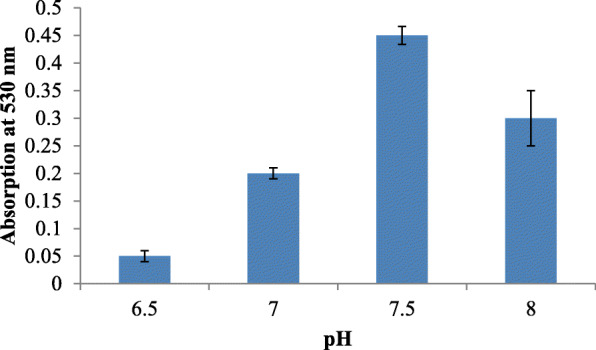
Effect of pH on prodigiosin production

**Fig. 2:**
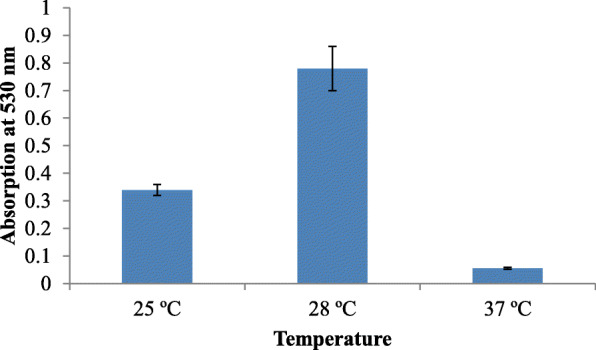
Effect of temperature on prodigiosin production

**Fig. 3:**
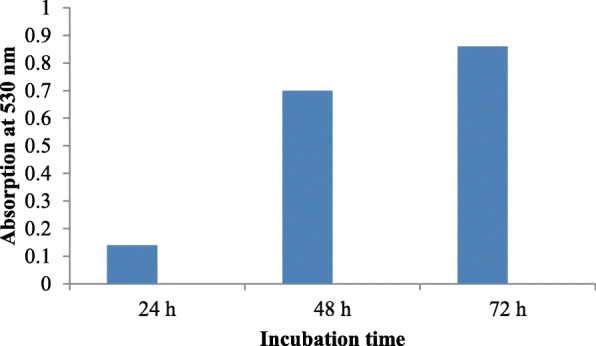
Effect of incubation time on prodigiosin production

**Fig. 4:**
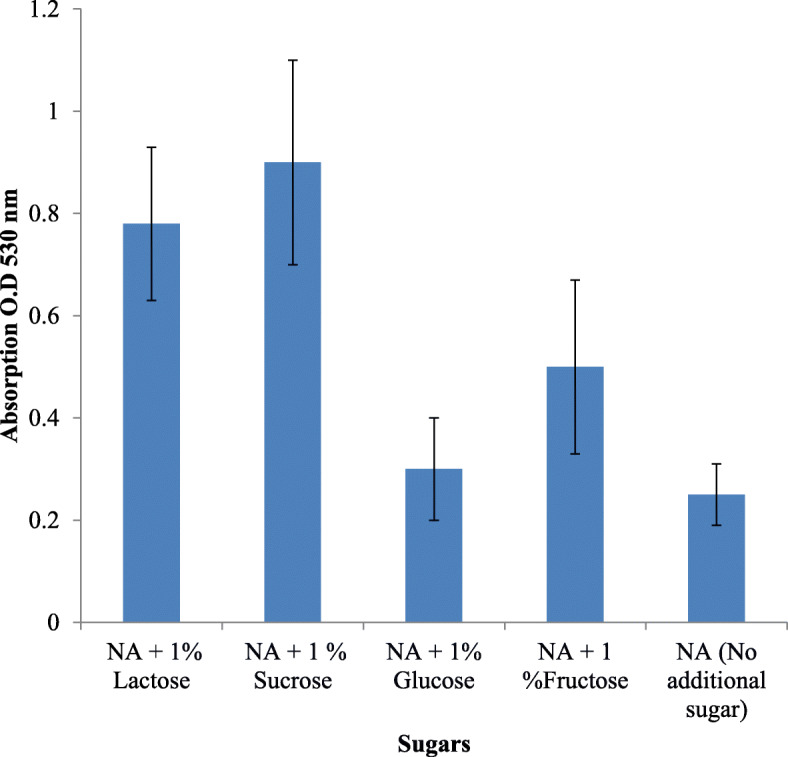
Effect of carbon source on prodigiosin production

**Fig. 5:**
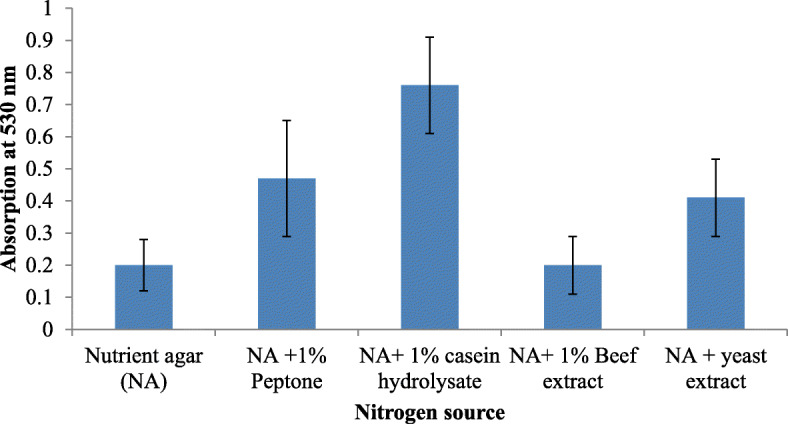
Effect of nitrogen source on prodigiosin production

**Fig. 6:**
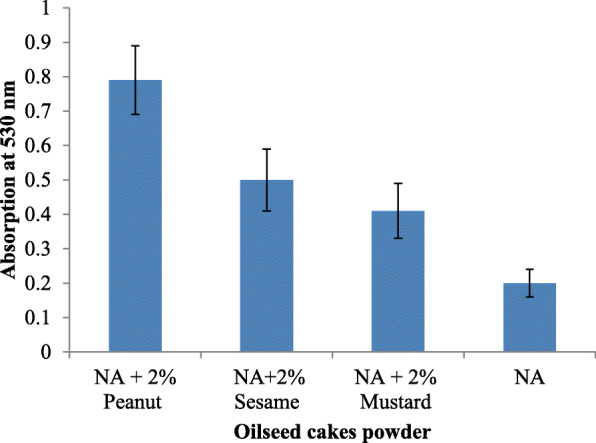
Effect of nutrient agar supplemented with different oil seed cake powders on prodigiosin production

**Fig. 7:**
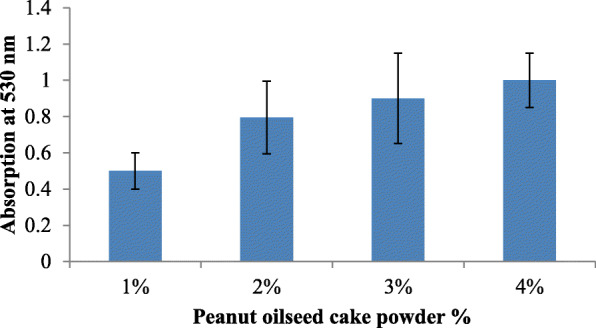
Effect of nutrient agar supplemented with peanut seed oilcake powder on prodigiosin production

**Fig. 8:**
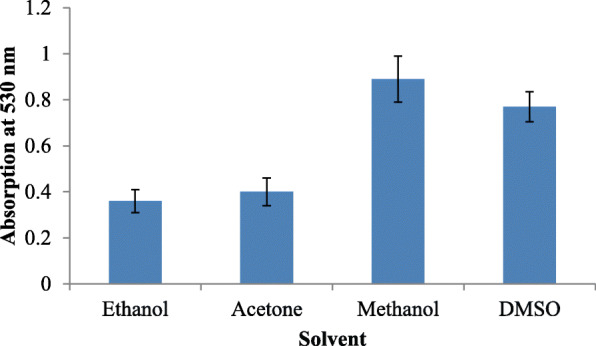
Effect of solvents on prodigiosin production

**Fig. 9:**
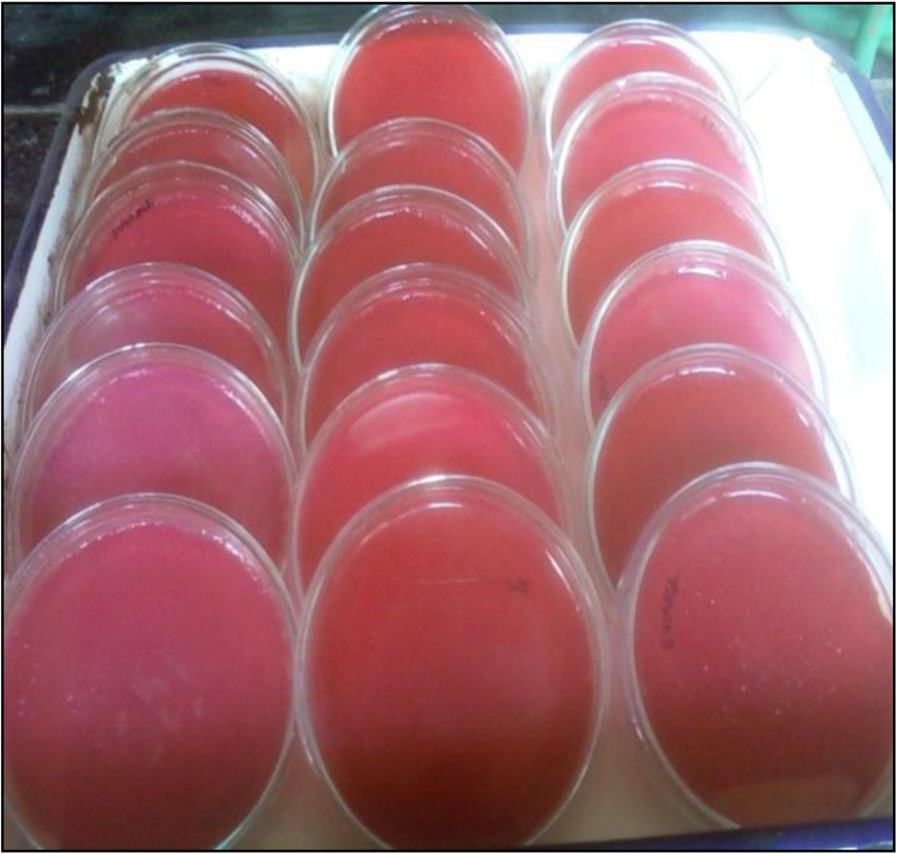
Pigment production on Petri plates

**Fig. 10:**
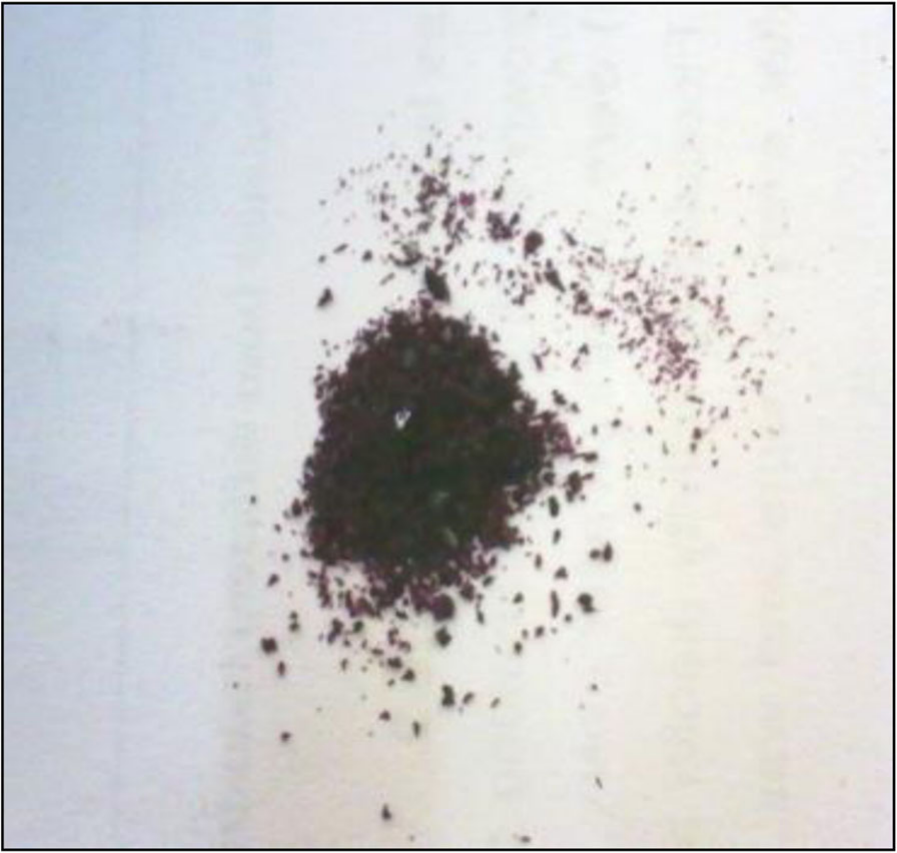
Crude extract dry powder

Growth on nutrient agar (0.6 mg/ml) resulted in higher pigment production than nutrient broth (0.35 mg/ml). The pigment was produced and purified on lab-scale using a solid optimized medium. Table 1 shows the optimized parameters suitable for enhanced prodigiosin production. Also, setting up parameters, like pH 7.5, temperature 28–30 °C, and 72 h incubation time, gave an enhanced pigment production (Figs. 1, 2, and 3). Sucrose and lactose revealed as better carbon sources for optimum prodigiosin production (Fig. 4). One percent casein hydrolysate revealed higher pigment production as compared to peptone, beef extract, and yeast extract supplements (Fig. 5). Peanut oil seed cake powder supplemented nutrient agar gave maximum production of pigment (Fig. 6). Moreover, 4% peanut oil seed cake powder concentration served as rich source of nutrients helping in raised amount of prodigiosin production (Fig. 7). The incorporation of optimized sucrose, lactose, and peanut oil seed cake powder, and other essential parameters resulted in maximum yield (3.5 mg/ml) of prodigiosin. Methanol was found to be the best solvent for extraction process (Fig. 8). The wet weight of the biomass produced was found to be 15.5 g/L (Fig. 9). The pigment was extracted and dissolved in minimum amount of methanol (50 ml) and dried using rotary evaporator at 60 °C. The dried powder obtained weighed 0.9 g/L (Fig. 10).

**Table 1:** Summary of optimized parameters for prodigiosin production

Sr. no.	Parameters	Optimized parameter	Concentration of pigment (mg/ml)
1	pH	7.5	0.48
2	Temperature	28 °C	0.45
3	Incubation time	72 h	0.85
4	Carbon source	2% Sucrose	2.4
5	Nitrogen source	1% casein hydrolysate	0.87
6	Fatty acid source	2% peanut oil seed cake powder 4% peanut oil seed cake powder	2.6 3.0
7	Optimized medium	2% Sucrose + 1% casein hydrolysate + 4% peanut oil seed cake powder, pH 7.5, temp. 28 °C, incubation time 72 h	3.5
7	Solvent	Methanol	0.95

**Fig. 11:**
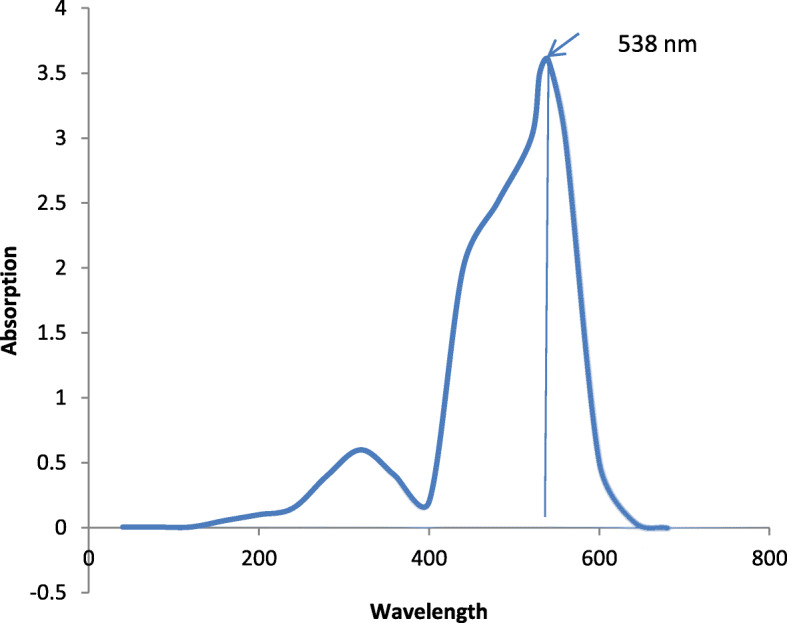
UV-scan image of methanolic extract

**Fig. 12:**
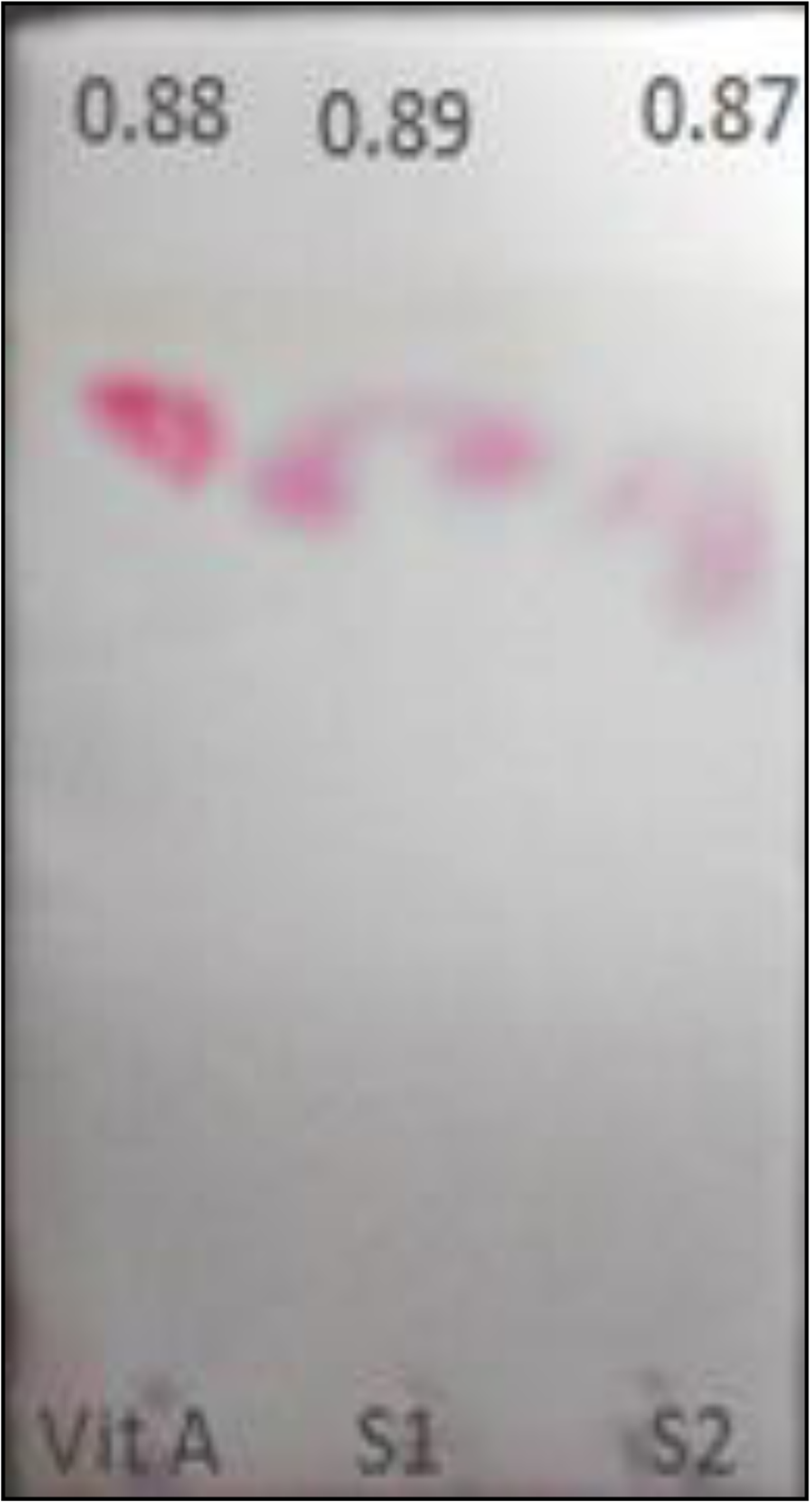
Chromatographic analysis by TLC. (Reference; vitamin A, S1; sample of methanolic extract of prodigiosin, S2; DMSO extract of prodigiosin)

The UV-scan results showed that the maximum absorbance of methanolic-dissolved extract was 538 nm (Fig. 11) while DMSO extract showed 539 nm. The pigment was characterized and confirmed by TLC (Fig. 12). The *R*_f_ value of methanolic extract was 0.89 and that of DMSO extract was 0.87. The GC-MS analysis revealed the molecular mass as 323 amu and structure was observed as 4-methoxy-5(5-methyl-4-pentyl-2H—pyrrol-2-ylidene)—2,2-bipyrrole, a structure similar to homologues of prodigiosin derivatives.

## Discussion

### Optimisation of culture growth conditions for prodigiosin biosynthesis

Microbial pigments are biologically active and medicinally important compounds released by some bacteria. Different media components should be analyzed and compared effectively to deduce the most probable reason for the enhancement or decline in pigment production. The parameters, like carbon source, nitrogen source, fatty acid source, pH, incubation time, temperature, and solvent, were optimized to obtain maximum production of prodigiosin. Keeping this in mind, a media which could support growth of bacteria and at the same time prove efficient to trigger high levels of pigment formation was designed. As the pigment was highly hydrophobic, it could not produce much pigment in liquid medium, solid media was used for its production. Rosenberg et al. have also mentioned about the cell surface hydrophobicity in their study [21].

While considering the basic role of carbon source in augmenting pigment production, the addition of sucrose and lactose was shown to enhance the pigment yield. The optimum pH for pigment production was determined to be 7.5 (Fig. 6). The bacterial isolate elaborated pigment production at 28–30 °C, and the rate decreased as temperature increased. The pigment production increased after incubation for 72 h while it decreased after 96 h suggesting that bacteria used the prodigiosin which is a protein as nutrition during stress conditions. Williams and Hussain Quadri had also reported that no pigment was produced when cultures were incubated at 37 °C [22].

Several researchers have screened various differential and selective media containing specific carbon sources for enhancing the pigment production. Capryllate thallous agar is one such media which has capryllate as carbon source and thallous salts to avoid contamination. Nakamura and his colleagues have reported use of regular media, like nutrient broth and peptone glycerol broth including 2% sodium oleate which resulted in enhanced pigment production of 0.69 mg/ml [23].

Giri et al. has reported the use of powdered sesame seeds in nutrient broth and peptone glycerol media for augmenting growth of *S. marcescens*. The researchers observed that sesame seeds resulted in higher yields of prodigiosin production as compared to other cheaper sources, like peanut and coconut. The researchers screened media for growth at varied temperature parameter for prodigiosin production. They suggested that fatty acids play an important role as a substrate for higher pigment production. It has been observed that various components in the seeds can stimulate the cell density which in turn resulted in higher concentration of positive regulators inside the cell thus triggering excessive pigment production. The maximum pigment was produced at 28 °C and 30 °C in nutrient broth. It was also observed that crushed sesame seed broth resulted in highest pigment production [1]. Similarly, in our study, incorporation of 4% peanut oil seed cake powder in nutrient agar resulted in maximum pigment production (3 mg/ml) at 28 °C after incubating for 72 h.

Cang et al. has reported a medium with ethanol and carbon source resulting in yield up to 3 mg/ml. In his studies, nutrient broth with glucose exhibited a two-fold increase of pigment production at 28 °C. But again, it was observed that enhanced pigment was found in sesame seed broth even without the addition of any sugars. In fact, they observed reduction in the pigment production in sesame seed medium with maltose at 28 °C as compared to only powdered sesame seed broth. The glucose sugar within powdered sesame seed medium showed a radical decrease of pigment at both 28 °C and 30 °C. The results suggested that maltose was a better source of substrate in enhancing pigment production but in nutrient broth [24]. The reason for reduction in pigment release in case of added glucose or maltose might be attributed to the catabolite repression mechanism. Researchers have observed that the addition of glucose to a rich and minimal media inhibited prodigiosin production. The cyclic AMP and CRP regulation of the flagellum were found to be responsible for the secondary metabolite production in *S. marcescens* strain PIC3611 [25].

In one study, when triolein an unsaturated fatty acid was substituted in the medium, a maximum yield of 0.69 mg/ml of pigment was recovered. Kim et al. in their study also observed that oil gave a better yield over other powdered oil seeds as carbon and nitrogen sources tested. They suggested that the bonded fatty acids as carbon source might less accessible by the organism [26].

Shahitha et al. in their study screened a similar fatty acid containing substrate called copra (coconut) which has the highest amount of fatty acids followed by peanut and sesame. But, in contrast to other studies, the pigment production was seen to be lesser in the presence of copra. From this fact, they deduced that the presence of 50% lauric and 7% capric acid which is anti-bacterial in nature might have inhibited the growth of *Serratia*. Peanut oil seed cake is a by-product obtained after the extraction of oil. Tekrony have revealed that peanut oil seed cake composition includes 45–60% protein, 22–30% carbohydrate, 4–6% minerals, and 3.8–7.5% crude fibre [27]. Also, peanuts have higher concentration of fatty acids than in sesame seeds and that may result in comparative enhanced pigment production than in sesame seeds as carbon substrate media [28].

After studying the literature of the composition of published media, there was a need for designing an innovative, nutrition-packed, and economically cheap medium for enhanced prodigiosin biosynthesis. The suitable fatty acid for pigment production was optimized by cultivating the bacteria in thepresence of 2% peanut seeds, sesame seeds, and mustard seeds oilcakes. The powdered peanut oil seed cake gave the highest yield of 3 mg/ml, and this result was compared with existing literature that used different carbon source. This medium is a rich source of minerals, vitamins, proteins saturated, and unsaturated fatty acids. *S. marcescens* is capable of producing a lipolytic enzyme or lipase which has ability to hydrolyse fatty acids in order to use as a carbon source [1].

### Confirmation and characterization of the pigment

The pigment separation and its *R*_f_ value was determined by thin layer chromatography method using silica gel plates. Vitamin A which is an analog of prodigiosin having pyrrole ring system and also similar molecular weight 333 amu was used as a standard. Two sample extracts in methanol(*R*_f_ = 0.87) and DMSO (0.89) separately were spotted and showed the same *R*_f_ value as that vitamin A (0.88) and that of prodigiosin orange red fraction (0.9) as given in literature [29]. Similar study was conducted by Someya and his colleagues who tested the prodigiosin content by scrapping off bacterial cells from Luria Bertanni agar plates and by suspending it in 9 ml of ethanol. The centrifuged cell pellets were fractionated by thin layer chromatography. The *R*_f_ value was then calculated and found to be in the range if 0.9–0.95 which is characteristic of the prodigiosin pigment [30].

The absorbance value of prodigiosin was determined by running an UV-wavelength scan. Prodigiosin pigment has a characteristic absorbance at 537 nm. Two 3 ml sample extracts each were dissolved separately in methanol and DMSO was scanned. Methanol extract showed maximum absorbance at 538 nm while DMSO extract showed absorbance at 539 nm. These results were similar to results obtained by Williams et al. [29]. Also, Kim et al. in his study has reported that prodigiosin recovered from *Serratia* spp. KH-95 had a maximum absorption spectrum at 535 nm [31].

The mass spectrophotometry analysis showed that compound obtained was 4-methoxy-5(5-methyl-4-pentyl-2H—pyrrol-2-ylidene)—2,2-bipyrrole ring structure. The mass calculated by GC-MS was found to be around near about 323 m/z. In a similar study conducted by Silva et al., the red pigment produced by *S. marcescens* had an absorbance of 534 nm and molecular weight equal to 323 m/z and was characterized as prodigiosin. Similarly, Yang et al. also reported a prodigiosin produced by *Microcystis aeruginosa* that had a molecular weight of 323 m/z [32].

## Conclusion

Prodigiosin yield was enhanced under optimized conditions. Using oil seed cakes proved to be economical and gave higher yield of prodigiosin. The optimized media especially with 4% peanut oil seed cake powder supplemented nutrient agar gave maximum prodigiosin production. The oil seed cake is known to be power packed with nutrients that might have led to a boost in the pigment production. This promising pigment is known to possess wide medicinal applications due to its anti-proliferative, anti-bacterial, and anti-mycotic activities. Hence, further study will focus on the medical applications of prodigiosin.

## Data Availability

Not applicable.

## Abbreviations

AR: Analytical reagent
LB: Luria Bertanni
NB: Nutrient broth
NA: Nutrient agar
D/W: Distilled water
HCl: Hydrochloric acid
NaCl: Sodium chloride
DMSO: Dimethyl sulfoxide
*R*_f_: Retardation factor
amu: Atomic mass unit

## References

[1] Giri AV, Anandkumar N, Muthukumaran G, Pennathur G (2004) A novel medium for the enhanced cell growth and production of prodigiosin from Serratia marcescens isolated from soil. BMC Microbiol. 10.1186/1471-2180-4-11 10.1186/1471-2180-4-11 PMC404375 15113456

[2] Bennett JW, Bentley R (2000) Seeing red: The story of prodigiosin. Adv Appl Microbiol. 10.1016/s0065-2164(00)47000-0 10.1016/s0065-2164(00)47000-0 12876793

[3] Han SB, Kim HM, Kim YH, Lee CW, Jang ES, Son KH et al (1998) T-cell specific immunosuppression by prodigiosin isolated from Serratia marcescens. Int J Immunopharmacol. 10.1016/S0192-0561(97)00062-3 10.1016/s0192-0561(97)00062-3 9717078

[4] Montaner B, Navarro S, Piqué M, Vilaseca M, Martinell M, Giralt E et al (2000) Prodigiosin from the supernatant of Serratia marcescens induces apoptosis in haematopoietic cancer cell lines. Br J Pharmacol. 10.1038/sj.bjp.0703614 10.1038/sj.bjp.0703614 PMC1572367 11015311

[5] Berlanga M, Ruiz N, Hernandez-Borrell J, Montero T, Vinas M (2000) Role of the outer membrane in the accumulation of quinolones by Serratia marcescens. Can J Microbiol. 10.1139/w00-052 10941517

[6] Sang BH, Se HP, Young JJ, Young KK, Hwan MK, Kyu HY (2001) Prodigiosin blocks T cell activation by inhibiting interleukin-2Rα expression and delays progression of autoimmune diabetes and collagen-induced arthritis. J Pharmacol Exp Ther 11602650

[7] Xia Y, Wang G, Lin X, Song X, Ai L (2016) Solid-state fermentation with Serratia marcescens Xd-1 enhanced production of prodigiosin by using bagasse as an inertia matrix. Ann Microbiol. 10.1007/s13213-016-1208-4

[8] Aniyan NB, Thomas SK (2019) Solid state fermentation for prodigiosin production using Serratia marcescens. Int Res J Eng Technol

[9] Fürstner A (2003) Chemistry and biology of roseophilin and the prodigiosin alkaloids: A survey of the last 2500 years. Angew Chemie - Int Ed. 10.1002/anie.200300582 10.1002/anie.200300582 12916029

[10] Yamashita M, Nakagawa Y, Li H, Matsuyama T (2001) Silica Gel-Dependent Production of Prodigiosin and Serrawettins by Serratia marcescens in a Liquid Culture. Microbes Environ. 10.1264/jsme2.2001.250

[11] Goldschmidt MC, Williams RP. Thiamine-induced formation of the monopyrrole moiety of prodigiosin. J Bacteriol 1968. doi:10.21123/bsj.4.4.622-627. 10.1128/jb.96.3.609-616.1968 PMC252349 4895047

[12] Witney FR, Failla ML (1977). Weinberg ED.

[13] Lawanson AO, Sholeye FO (1976) Inhibition of prodigiosin formation in Serratia marcescens by adenosine triphosphate. Experientia. 10.1007/BF01920782 10.1007/BF01920782 773662

[14] De Araújo HWC, Fukushima K, Takaki GMC (2010) Prodigiosin production by Serratia marcescens UCP 1549 using renewable-resources as a low cost substrate. Molecules. 10.3390/molecules15106931 10.3390/molecules15106931 PMC6259207 20938403

[15] Xia S, Veony E, Yang Q (2018) Kitchen waste as a novel available substrate for prodigiosin production by Serratia marcescens. IOP Conf. Ser. Earth Environ. Sci. 10.1088/1755-1315/171/1/012037

[16] Gutiérrez-Román MI, Holguín-Meléndez F, Bello-Mendoza R, Guillén-Navarro K, Dunn MF, Huerta-Palacios G (2012) Production of prodigiosin and chitinases by tropical Serratia marcescens strains with potential to control plant pathogens. World J Microbiol Biotechnol. 10.1007/s11274-011-0803-6 10.1007/s11274-011-0803-6 22806790

[17] Wei YH, Chen WC (2005) Enhanced production of prodigiosin-like pigment from Serratia marcescens SMAR by medium improvement and oil-supplementation strategies. J Biosci Bioeng. 10.1263/jbb.99.616 10.1263/jbb.99.616 16233840

[18] Sunil L, Appaiah P, Prasanth Kumar PK, Gopala Krishna AG (2015) Preparation of food supplements from oilseed cakes. J Food Sci Technol. 10.1007/s13197-014-1386-7 10.1007/s13197-014-1386-7 PMC4397353 25892801

[19] Bae J, Moon H, Oh KK, Kim CH, Sil Lee D, Kim SW, et al. A novel bioreactor with an internal adsorbent for integrated fermentation and recovery of prodigiosin-like pigment produced from Serratia sp. KH-95. Biotechnol Lett 2001. doi:10.1023/A:1010573427080.

[20] Khanafari A, Assadi MM, Fakhr FA (2006) Review of prodigiosin, pigmentation in Serratia marcescens. Online J Biol Sci. 10.3844/ojbsci.2006.1.13

[21] Rosenberg M, Blumberger Y, Judes H, Bar-Ness R, Rubinstein E, Mazor Y (1986) Cell surface hydrophobicity of pigmented and nonpigmented clinical Serratia marcescens strains. Infect Immun. 10.1128/iai.51.3.932-935.1986 10.1128/iai.51.3.932-935.1986 PMC260988 3512440

[22] Qadri SM, Williams RP (1972) Induction of prodigiosin biosynthesis after shift-down in temperature of nonproliferating cells of Serratia marcescens. Appl Microbiol. 10.1128/aem.23.4.704-709.1972 10.1128/am.23.4.704-709.1972 PMC380422 4553139

[23] Nakamura A, Nagai K, Ando K, Tamura G (1986) Selective suppression by prodigiosin of the mitogenic response of murine splenocytes. J Antibiot (Tokyo). 10.7164/antibiotics.39.1155 10.7164/antibiotics.39.1155 2944863

[24] Cang S, Sanada M, Johdo O, Ohta S, Nagamatsu Y, Yoshimoto A (2000) High production of prodigiosin by Serratia marcescens grown on ethanol. Biotechnol Lett. 10.1023/A:1005646102723

[25] Kalivoda EJ, Stella NA, Aston MA, Fender JE, Thompson PP, Kowalski RP et al (2010) Cyclic AMP negatively regulates prodigiosin production by Serratia marcescens. Res Microbiol. 10.1016/j.resmic.2009.12.004 10.1016/j.resmic.2009.12.004 PMC2846241 20045458

[26] Kim CH, Kim SH, Hong SI. Isolation and characteristics of prodigiosin-like red pigment produced by Serratia sp. KH-95. Korean J Appl Microbiol Biotechnol 1998.

[27] TeKrony DM (2005) Seeds Handbook: Biology, Production, Processing and Storage (second edition). Crop Sci. 10.2135/cropsci2005.0006

[28] Shahitha S, Poornima K (2012) Enhanced production of prodigiosin production in Serratia marcescens. J Appl Pharm Sci. 10.7324/JAPS.2012.2823

[29] Williams RP, Green JA, Rappo-Port DA. Studies on pigmentation of Serratia marcescens. I. Spectral and paper chromatographic properties of prodigiosin. J Bacteriol 1956. 10.1128/jb.71.1.115-120.1956 PMC357744 13286239

[30] Someya N, Nakajima M, Hamamoto H, Yamaguchi I, Akutsu K (2004) Effects of light conditions on prodigiosin stability in the biocontrol bacterium Serratia marcescens strain B2. J Gen Plant Pathol. 10.1007/s10327-004-0134-7

[31] Song MJ, Bae J, Lee DS, Kim CH, Kim JS, Kim SW, et al. Purification and characterization of prodigiosin produced by integrated bioreactor from Serratia sp. KH-95. J Biosci Bioeng 2006. doi:10.1263/jbb.101.157. 10.1263/jbb.101.157 16569612

[32] Yang F, Wei HY, Li XQ, Li YH, Li XB, Yin LH et al (2013) Isolation and characterization of an algicidal bacterium indigenous to lake taihu with a red pigment able to lyse Microcystis aeruginosa. Biomed Environ Sci. 10.3967/0895-3988.2013.02.009 10.3967/0895-3988.2013.02.009 23336138

